# Editorials

**Published:** 1911-03

**Authors:** 


### EDITORIALS

The Business Office of the *Journal-Record* is 1 1-2 to 5 1-2 South Broad Street.
The Editorial Office is 1014-15 Century Building.
Address all Business Communications to *Journal-Record of Medicine*, 1 1-2 to 5 1-2 South Broad Street.
Make remittances payable *to The Journal-Record of Medicine*.
On matters pertaining to the Editorial and Original Communications, address Edgar G. Ballenger, M. D., Atlanta, Ga.
Reprints of Original Articles will be furnished at cost price. Requests for the same should always be made in THE MANUSCRIPT.
We will present, postpaid, on request, to each contributor of an original article, twenty (20) marked copies of *The Journal-Record of Medicine* containing such article.

### THE PUBLIC SCHOOL CURRICULUM.

Before this is printed in our columns we sincerely hope the much-talked-of "Nutting resolution," or rather absurdity, will be voted down by the city council. This resolution seeks to abolish from the curriculum of the city schools art, music, physical culture and hygiene. Not being familiar with the politics of the situation, the writer is unable to say just what is the reason of such a move, but probably the alleged cause is a political or financial one.

The average thoughful individual must realize that our schools are now already far from what they should be and such a step would be backward, not forward, and would be a lasting disgrace to Atlanta. The question of schools is undoubtedly one needing reform, radical reform, but not regressive, but progressive reform. Many pertinent questions might be asked regarding the advisability and usefulness of certain features of the curriculum, but the present resolution is one far too absurd to receive favorable action on the part of an intelligent body of men, who must realize the folly of crowding a child's head full of many things of doubtful value at the expense of curtailing the training in hygiene, physical culture, etc. What could be of greater value to the individual and to the state than proper instruction as to eating, breathing, sleeping, walking, brushing of teeth, cleanliness, and all these little things regarding bodily care? If there is a single academic branch, or course even, that can compare in importance with these things the writer does not know what it is.

The usefulness of art and music in making the homes more attractive and the individuals more contented cannot be denied. The actual physical training is more needed than the average academic study. Who can doubt the value of anything in the curriculum which tends to refine and cultivate the children?

---

### CHATTAHOOCHEE VALLEY MEDICAL AND SURGICAL ASSOCIATION.

---

The Chattahoochee Valley Medical and Surgical Association held its ninth Semi-Annual Session in the parlors of the Racine Hotel, on January 10-11, 1911. There were present fortv physicians from Alabama and Georgia, and eight new members were added to the roll at this meeting.

This being the time for election of new officers, the following were elected: Dr. L. W. Johnson, Tuskegee, Alabama, President; H. M. Lokey, Atlanta, Ga., 1st Vice-President; T. H. Haralson, Cusseta, Ala., 2nd Vice-President.

Program Committee: R. S. Hill, Montgomery, Ala.; W. J. Love, Opelika, Ala.; Geo. M. Niles, Atlanta, Ga.; L. W. Johnston, Tuskegee, Ala.

The Board of Council: Geo. M. Niles, J. H. McDuffie, Geo. H. Cooper, A. L. Harlan and H. B. Disharoon.

At this meeting it was decided to change the time of meeting from the second Tuesday and Wednesday of January and July, to the third Tuesday and Wednesday of January and July, as the former dates conflicted with so many other organizations in which the doctors were intersted, it was thought best, and the vote was unanimous for the change.

Tuskegee was selected as the next meeting place of the Association and the date the third Tuesday and Wednesday in July, 1911.

Columbus sustained her reputation for hospitality, and the banquet tendered the doctors was voted delightful in every respect, in fact, all visiting doctors left singing the praises of Columbus and her excellent body of physicians and surgeons.

